# CADASIL from Bench to Bedside: Disease Models and Novel Therapeutic Approaches

**DOI:** 10.1007/s12035-021-02282-4

**Published:** 2021-01-19

**Authors:** Arianna Manini, Leonardo Pantoni

**Affiliations:** Stroke and Dementia Lab, “Luigi Sacco” Department of Biomedical and Clinical Sciences, University of Milan, Via Giovanni Battista Grassi 74, 20157 Milano, Italy

**Keywords:** CADASIL, *NOTCH3*, Transgenic mice, iPSC, Antisense oligonucleotides, Immunotherapy

## Abstract

Cerebral autosomal dominant arteriopathy with subcortical infarcts and leukoencephalopathy (CADASIL) is a monogenic disease caused by *NOTCH3* mutations and characterized by typical clinical, neuroradiological, and pathological features. NOTCH3 belongs to a family of highly conserved transmembrane receptors rich of epidermal growth factor repeats, mostly expressed in vascular smooth muscle cells and pericytes, which perform essential developmental functions and are involved in tissues maintenance and renewal. To date, no therapeutic option for CADASIL is available except for few symptomatic treatments. Novel in vitro and in vivo models are continuously explored with the aim to investigate underlying pathogenic mechanisms and to test novel therapeutic approaches. In this scenario, knock-out, knock-in, and transgenic mice studies have generated a large amount of information on molecular and biological aspects of CADASIL, despite that they incompletely reproduce the human phenotype. Moreover, the field of in vitro models has been revolutionized in the last two decades by the introduction of induced pluripotent stem cells (iPSCs) technology. As a consequence, novel therapeutic approaches, including immunotherapy, growth factors administration, and antisense oligonucleotides, are currently under investigation. While waiting that further studies confirm the promising results obtained, the data reviewed suggest that our therapeutic approach to the disease could be transformed, generating new hope for the future.

## Introduction

Cerebral autosomal dominant arteriopathy with subcortical infarcts and leukoencephalopathy (CADASIL) is a monogenic disease caused by *NOTCH3* mutations [1]. Age of onset is variable from infancy to elderly, even though CADASIL patients usually develop first symptoms during early adulthood [2]. Main clinical aspects include migraine typically with aura [3], recurrent subcortical ischemic strokes and transient ischemic attacks presenting as lacunar syndromes [4], apathy and a number of psychiatric disturbances [5, 6], cognitive impairment [7], and acute encephalopathy [8]. Neuroimaging features, as detected by magnetic resonance imaging (MRI), are supportive of the diagnosis even 10–15 years before the onset of clinical manifestations and consist of white matter hyperintensities (WMH), lacunes, microbleeds, and dilated perivascular spaces, as in many other small vessel diseases (SVDs) [9]. WMH on T2-weighted or fluid-attenuated inversion recovery might be detected in the first phases of disease only in periventricular areas and in the centrum semiovale; subsequently, they spread symmetrically involving also the external capsule, the anterior portion of the temporal lobes, and the superior frontal gyrus [10].

CADASIL represents a monogenic disease with autosomal dominant inheritance caused by mutations of *NOTCH3* which maps on chromosome 19p13.1 [11]. *NOTCH3*, a large gene containing 33 exons, encodes for a single-pass transmembrane receptor rich of epidermal growth factor repeats (EGFRs) in the extracellular domain, expressed in vascular smooth muscle cells (VSMCs) and pericytes [12, 13]. More than 200 *NOTCH3* pathogenic variants have been described in CADASIL families so far. Over 95% of them are represented by missense mutations involving a cysteine residue in one of the EGFRs, mostly located in exons 3 and 4 [11], even though they can be found in all EGFR coding exons (2–24) [14]. The consequence is an increased number of cysteine residues in one of the 34 EGFR domains and the presence of one unpaired cysteine residue, resulting in an incomplete disulfide bridge with an increased NOTCH3 ectodomain (NOTCH3^ECD^) multimerization potential [15]. *NOTCH3* mutations lead to VSMCs degeneration that is accompanied by the accumulation of granular osmiophilic material (GOM) in the extracellular space surrounding VSMCs, detected by electron microscopy [16]. Even though the whole composition of GOM is still debated, studies showed that NOTCH3^ECD^ represents a significant constituent of these deposits [17]. Although VSMCs degeneration represents a pathological hallmark of CADASIL, an increasing number of studies has showed a significant role of endothelial cells in its pathogenesis. Endothelial cell injury seems to take part in vessel thrombosis, resulting in blood flow decrease, endothelial cell functional impairment, blood–brain barrier disruption, and, ultimately, brain parenchyma damage [18, 19]. Even though the monogenic cause differentiates it from sporadic SVDs, CADASIL represents a unique opportunity to investigate features of SVDs. An effective therapy for CADASIL is still not available, but recent findings deriving from experimental models might help considerably in this direction.

The purpose of this review is to provide an update of in vitro and in vivo models of CADASIL and to illustrate the most recent experimental therapeutic approaches currently under investigation.

## Disease Models

Different disease models have been developed to investigate CADASIL pathogenic mechanisms and to assess the safety and effectiveness of novel therapeutic strategies. Here, we provide a review of the most relevant in vivo and in vitro disease models realized over the last decades.

### In Vivo Models

#### Drosophila Lethal-Abruptex

NOTCH3 belongs to an evolutionarily conserved family of transmembrane receptors which includes *Drosophila melanogaster* Notch homologs 1 to 4 [20]. *Drosophila* Abruptex alleles represent a specific category of mutations within the *Drosophila Notch* gene associated with a typical phenotype, which includes interrupted wing veins and a defective number of dorsal bristle; this phenotype is due to amino acid substitutions within the extracellular domain of the Notch receptor, affecting the binding affinity for ligands, including Delta [21–25]. Among this group of mutations, *Drosophila* lethal-Abruptex resembles main features of CADASIL *NOTCH3* variants by altering the number of cysteines in EGFR domains. It seems reasonable that the *Drosophila* lethal-Abruptex gene product inhibits Notch signaling [26] by interacting with the normal gene product at the cell surface and impairing the secondary structure of its extracellular domain so that it cannot cross-link adequately to binding proteins. Even though *Drosophila* lethal-Abruptex represents an in vivo model of CADASIL due to molecular similarities, *Drosophila* owns only one *Notch* gene, while mammals have four *NOTCH* genes [27], each one showing tissue-specific expression, thus producing significant differences between Abruptex and CADASIL.

#### Zebrafish *Notch3* Mutants

Zebrafish transgenic models have been successfully generated. Specifically, Zaucker et al. [28] analyzed the effects of two mutations in the zebrafish *Notch3* gene: the *st51*, which is known to reduce the expression of myelin basic protein [29], thus decreasing the number of oligodendrocyte precursor cells in larvae; the second mutation consisted of an insertional allele produced by a retroviral insertion. Histological studies performed on the *Notch3* homozygous mutants survived to adulthood and, on adult zebrafish carrying both mutant alleles, showed impairment of vascular structure and function, including dilated and disorganized vessels and gaps in the arterial wall which produced hemorrhages, in association with reduced expression of Notch3 target genes such as *hey1*. On the other side, the presence of high *Notch3* and *hey1* expression in heterozygotes demonstrated the occurrence of modification in gene expression after incomplete loss of Notch3 [28]. Unfortunately, mutant *Notch3* zebrafish does not recapitulate completely CADASIL pathology, thus highlighting the need for further models.

#### *NOTCH3* Knock-Out Mice

Although *NOTCH3* knock-out (NOTCH3KO) mice do not represent a genetic model of CADASIL, they provide essential evidence of NOTCH3 role in cerebrovascular maturation and homeostasis. Homozygous NOTCH3KO mice, despite being viable, fertile, and presenting no patent anatomic aberrations [30], display abnormalities of differentiation in small systemic and cerebral resistance arteries. Structural arterial deficits recorded in homozygous NOTCH3KO mice include a venous pattern of maturation and an altered location and orientation of VSMCs in the tunica media and around the lumen of small arteries due to compromised remodeling of their final shape [31]. Arboleza-Velasquez et al. [32] failed to detect the same aberrations in brain vessels and aorta of homozygous NOTCH3KO mice but found a downregulation of the conventional NOTCH3 downstream targets appointed to muscle contraction and maturation. Another work [33] showed that NOTCH3 deficiency produces functional deficits exclusively in cerebral small and tail caudal arteries, with a severe impairment of myogenic tone, thus compromising autoregulation of cerebral blood flow. Nevertheless, the lack of myogenic response restricted to isolated cerebral and tail arteries does not affect systemic blood pressure, which appears regulated mostly by larger peripheral vascular beds and hormonal vasoactive systems, including the sympathetic and renin-angiotensin systems [33]. Increased ischemia susceptibility was demonstrated in NOTCH3KO mice when occluding the proximal middle cerebral artery, which produced more than twofold larger infarcts compared to both *NOTCH3* wild type (WT) and heterozygous NOTCH3KO mice [32]. The expression of WT *NOTCH3* in VSMCs rescued the ischemic phenotype of NOTCH3KO mice, thus confirming the role of NOTCH3 in VSMCs ischemic susceptibility [32].

#### CADASIL Mutant Mice

Several CADASIL mutant mouse models have been realized and analyzed over the last decades [34–37]. Main differences among these models involve transgenic strategy, levels of expression of mutant *NOTCH3*, expression of endogenous *NOTCH3*, and consequences on NOTCH3 function.

The progressive development of arterial defects was analyzed by Ruchoux et al. [37] in a transgenic mouse model expressing the R90C mutation in the *NOTCH3* human gene (TgNotch3^R90C^), an archetypal CADASIL mutation located in the EGFR2 and responsible for the addition of a cysteine residue. TgNotch3^R90C^ mice were developed through a smooth muscle-specific promoter (SM22α), which drives the expression of a full-length human *NOTCH3* cDNA carrying the R90C mutation in VSMCs and pericytes. The presence of signs of VSMCs degeneration, such as cytoskeleton changes and defective anchorage to extracellular matrix and cells before the deposition of NOTCH3^ECD^ and GOM accumulation, suggests that these deposits might not be the trigger of VSMCs deterioration [37]. The functional consequences of the disruption of adhesion of VSMCs to the surrounding micro-environment consist of an impaired myogenic response to shear and tensile stress, while agonist or receptor-induced tone remains unchanged [38]. The increased actin polymerization in VSMCs might be the responsible of higher myogenic tone of arteries in this model; on the other side, the altered flow-mediated dilation might result from an indirect effect on endothelial cells [37]. Furthermore, Lacombe et al. [39] described an impaired cerebral vasoreactivity consisting of decreased relaxation and increased resistance of cerebral arteries, probably due to VSMCs dysfunction rather than VSMCs degeneration. Direct consequences are an altered cerebral blood flow autoregulation and an increased susceptibility to hypotension, thus promoting ischemic events [39]. In another work, focused on pericytes in TgNotch3^R90C^ mice, their mitochondrial injury and autophagic degeneration were observed [40]. In TgNotch3^R90C^ mice, NOTCH3 activity was not altered and NOTCH3^ECD^ aggregates did not inhibit WT NOTCH3 function [41]. Actually, NOTCH3 activity levels remain constant in brain arteries of TgNotch3^R90C^ mice, especially RBP-Jκ-mediated signaling activity which regulates the transcription of target genes in response to the binding of ligands belonging to the Delta/Jagged family and expressed on surrounding cells [41]. WT NOTCH3 removal did not influence the burden of white matter lesions in TgNotch3^R90C^ mice [42]. Taken all together, these findings suggest that CADASIL pathogenesis might be associated with NOTCH3 gain of function rather that signaling loss.

Unlike TgNotch3^R90C^ mice, those expressing the C428S mutation in the *NOTCH3* human gene under the control of murine SM22α promoter (TgNotch3^C428S^) showed a loss of WT NOTCH3 activity and a mild dominant negative effect [36]. The authors [36] speculated that the consequences produced by the C428S mutation might be related to its location in the ligand-binding domain EGFR10, perhaps by the addition of an unpaired cysteine residue able to titrate WT *NOTCH3* receptor into non-active complexes, thus reducing the RBP-Jκ-mediated signaling activity. In both the last two mouse models (TgNotch3^R90C^ and TgNotch3^C428S^ mice), Monet-Lepretre et al. [43] demonstrated that NOTCH^ECD^ accumulation induces the abnormal recruitment of extracellular matrix proteins, including tissue inhibitor of metalloproteinases 3 (TIMP3) and vitronectin, whose dysregulation contributes to these aggregates’ toxicity on small vessels.

Contrary to the transgenic mice above mentioned, those expressing the R142C mutation of the *NOTCH3* mouse gene (TgNotch3^R142C^), corresponding to the very common human R141C, did not show the typical CADASIL pathological and neuroradiological features up to 20 months of age [35]. This mouse model was generated by employing a targeting construct containing the R142C for homologous recombination in embryonic stem cells (knock-in). The authors [35] excluded that the absence of CADASIL phenotype was due to an altered expression or processing of R142C *NOTCH3* at RNA or protein levels. They hypothesized that a species difference between human and murine *NOTCH3* could explain why mice carrying the murine gene mutated at the Arginine 142 site did not develop a CADASIL phenotype, unlike CADASIL patients with *NOTCH3* R141C mutation and TgNotch3^R90C^ mice, which carried the human *NOTCH3* mutated gene [35].

CADASIL pathological hallmarks, such as NOTCH3^ECD^ aggregations and GOM, were detected at younger age in the transgenic mice expressing the R169C mutation of *NOTCH3* rat gene (TgNotch3^R169C^) compared to previous models, respectively at 1–2 months and 5 months of age in TgNotch3^R169C^ mice [34] and at 14–16 months in TgNotch3^R90C^ ones [37]. The TgNotch3^R169C^ mice were developed by introducing the *NOTCH3* R169C mutation into a large P1-derived artificial chromosome (PAC) and showed a significantly higher expression of *NOTCH3* mutant gene [34] compared to previous models. Furthermore, the distribution of transgene expression resembled that of the endogenous gene. The authors reported the presence of early isolated white matter lesions and hypoperfusion not only in the areas of brain damage but also in those free from lesions [34], consistent with human findings [44]. Previous works showed a strong relation between hypoperfusion and white matter damage both in human and in mouse models [45–47]. In this model, the presence of significantly decreased resting cerebral blood flow, especially in white matter, even months before the appearance of brain damage, corroborates the hypothesis that hypoperfusion could be involved in white matter lesions pathogenesis [34]. The origin of severe and early reduction in cerebral blood flow is uncertain, but it might be secondary to decreased myogenic response, altered mechanical arterial functions, reduced vessel diameter resulting in increased vascular resistance, and capillary rarefaction [34]. The altered myogenic response was proved to be caused by abnormal hyperpolarization in parenchymal and pial arterioles secondary to an increased number of voltage-gated potassium (K_V_1) channels, which are involved in vascular smooth muscle membrane potential regulation, induced by disruption of TIMP3-sensitive pathway [48]. Indeed, TIMP3 inhibits the metalloprotease ADAM17 and HB-EGF, an EGFR agonist, in order to maintain cerebral vascular tone [49]. Reduced vasoconstriction secondary to increased luminal pressure was observed not only in pial arteries and cortical parenchymal arterioles [48, 49] but also in hippocampal microcirculation of TgNotch3^R169C^ mice [50]. HB-EGF, which restores normal depolarization of VSMCs membrane, rescued cerebrovascular reactivity [50]. Since TgNotch3^R169C^ mice developed NOTCH3^ECD^ accumulation, GOM deposition, and white matter lesions in the absence of infarcts or motor deficits [34], this model is considered suitable to reproduce the early pre-symptomatic phases of disease. Segmental intramyelinic edema, represented by microvacuoles with focal myelin degradation without oligodendrocyte loss and axonal injury, was suggested to be an early alteration of white matter in CADASIL [51]. TgNotch3^R169C^ mice showed also an impairment of NOTCH3 function in hippocampal precursor cells regulation, which depends on a balance between NOTCH1 and NOTCH3 binding to RBPJκ [52], producing respectively an activation or inhibition of hairy and enhancer of split (*HES*) and *HES*-related with YRPW motif (*HEY*) gene transcription [53]. Consistent with this evidence, an impairment in hippocampal neurogenesis at 12 months of age was described, probably due to an age-dependent suppression of cell proliferation by NOTCH3 over-expression [52]. Impaired hippocampal neurogenesis was confirmed both in TgNotch3^R169C^ and TgNotch3^WT^ by failure of environmentally enriched or running wheel cages, which simulate physiological neurogenesis stimuli [54]. Although reduced if compared with that of WT mice, physical activity of TgNotch3^R169C^ mice was higher [54] than the rate of running wheel activity required to induce neurogenesis [55]. In a following work, the R169C *NOTCH3* mutation was showed to be associated with decreased maximal dilation of cerebral arteries secondary to a higher *NOTCH3* activity [56]. These findings seem to differentiate the R169C *NOTCH3* mutation from those located in the ligand-binding domain of *NOTCH3*, which are predicted to reduce NOTCH3 activity and provide an explanation to the phenotype of R169C, which shows a more severe cognitive decline than the others according to genotype-phenotype correlation studies [36]. Furthermore, this transgenic mouse model was employed to confirm the role of endoplasmic reticulum stress and RhoA/Rho kinase in CADASIL pathogenesis, as proved by the normalization of vascular dysfunction through the employment of 4-PBA and fasudil, two pharmacological agents that inhibit endoplasmic reticulum stress and Rho kinase [57]. In this TgNotch3^R169C^ mice model, it was found a reduced number of pericytes and a decreased pericytes coverage of cortical vessels which causes blood–brain barrier disruption and plasma proteins extravasation [58].

*NOTCH3* transgenic mice expressing the mutated human *NOTCH3* transgene C455R and R1031C by Cre-mediated recombination (TgNotch3^R1031C^ and TgNotch3^C455R^ mice), inserted into the ROSA locus [59], were realized by using the SM22α promoter [60]. TgNotch3^R1031C^ and TgNotch3^C455R^ mice showed the expected pathological CADASIL features without developing ischemic events [60]. Even though the age-dependent development of deposits in transgenic mice resembles what happens in CADASIL patients, GOM localization is quite different. Indeed, it is found not only in the extracellular space near plasma membrane of VSMCs [16] but also inside cells, in association with vesicles and inclusions [60]. The R1031C and C455R mutations, firstly detected in Colombian kindreds, are located respectively in the EGFR26 and in the EGFR11, which contains a ligand-binding domain [61]. While the first has been associated with a typical onset in the fourth decade of life, the second one is carried by patients who usually develop ischemic events earlier [61]. Experiments on mouse embryonic fibroblasts expressing a NOTCH3 ligand and WT NOTCH3 or mutant NOTCH3 receptors were performed, supporting the hypothesis of an age-dependent hypomorphic phenotype, with a stronger loss of function mechanism associated with the C455R rather than with the R1031C mutation [60]. Intriguingly, proteomic analysis revealed that clusterin and collagen 18α1 (COL18A1), which is cleaved post translationally into the antiangiogenic endostatin, were highly expressed in CADASIL samples rather than controls and were detected in GOMs of CADASIL-affected arteries [60]. TgNotch3^R1031C^ and TgNotch3^C455R^ mice were subsequently analyzed to identify candidate CADASIL biomarkers such as increased plasma levels of COL18A1, endostatin and HtrA serine protease 1 (HTRA1), reduced levels of NOTCH3^ECD^, and loss of VSMC in retinal vessels [62]. The increased plasma levels of HTRA1 acquire an intriguing meaning if we consider that the *HTRA1* gene represents the genetic cause of cerebral autosomal recessive arteriopathy with subcortical infarcts and leukoencephalopathy (CARASIL) [63]. Even though CADASIL and CARASIL share similar clinical, neuroradiological, and pathological features [64], the role and the origin of HTRA1 accumulation in CADASIL remain uncovered.

TgNotch3^R182C^ mice, developed by using a BAC clone containing the human NOTCH3 gene and subsequently introducing the R182C variant by two-step Red-mediated recombination, showed progressively increasing age- and *NOTCH3* RNA expression level-dependent vascular accumulation of NOTCH3 and GOM deposits, which progress over time and are continuously being formed, without brain parenchyma lesions occurrence [65]. This finding led authors to realize the “NOTCH3 score” as quantitative biomarker for CADASIL, attempting to transform transgenic CADASIL mice in a proper model for pre-clinical testing of therapeutic approaches [66].

Clinical and pathological features of CADASIL were partially but not exhaustively reproduced by TgNotch3^R170C^ mice [67]. Since the age of 8 months only a minority of mice developed GOM deposition, VSMCs abnormalities with enlargement of intercellular spaces, microbleeds, thrombosis, fibrillar gliosis, microinfarctions, and neurological features, including ataxia and paresis [67]. TgNotch3^R170C^ mice did not show an altered Notch3 signaling [67], and expression levels of Notch3 target genes were not reduced when compared with WT mice [42], thus supporting the hypothesis that CADASIL pathogenesis might be secondary to a NOTCH3 gain of function rather that a signaling inhibition. Unlike previous models [36, 37], evidence of NOTCH3^ECD^ accumulation was detected only in one study [42], while others failed to confirm it [67]. These discoveries excluded that the lack of typical pathological findings usually found in mice could be explained by their short lifespan, which should have not allowed the development of cell degeneration. Perhaps, the significant differences of murine brain vessels compared to human ones might reduce their vulnerability to hypoperfusion, thus explaining the incomplete phenotype developed by mice. Nevertheless, their role in providing increasing knowledge about CADASIL pathogenesis and relevant features is irreplaceable.

Main features of knock-out, knock-in, and transgenic mouse models are summarized in Table 1.

**Table 1:** *NOTCH3* mouse models

Genetic background	Mutant transgene	Genetic manipulation	Promoter	Cloning vector	Transgene expression level (% of endogenous NOTCH3 mRNA)	Notch3^ECD^ aggregates	GOM	VSMCs abnormalities	White matter lesions	Lacunar infarcts	Clinical deficits	References
C57BL/6	*NOTCH3* null	KO			0	–	–	+	–	–	–	[30–33]
C57BL/6	WT human *NOTCH3*	Tg	SM22α		75%	–	–	–	–	–	–	[36, 37, 41, 43, 62, 65, 66]
FVB/N	WT rat *Notch3* (line 129)	Tg		PAC	400%	–	–	–	–	–	–	[34, 52, 56, 58]
C57BL/6	R90C human *NOTCH3*	Tg	SM22α		85%	+	+	+	–	–	–	[37–41, 43]
C57BL/6; 129/Sv	R142C mouse *Notch3*	K-in			100%	–	–	–	–	–	–	[35]
C57BL/6	C428S human *NOTCH3*	Tg	SM22α		150%	+	+	+	–	–	–	[36, 43]
FVB/N	R169C rat *Notch3* (line 92)	Tg		PAC	200%	+	+	+	–	–	–	[34, 42, 50, 52, 54, 58]
FVB/N	R169C rat *Notch3* (line 88)	Tg		PAC	400%	+	+	+	+	–	–	[34, 42, 50, 52, 54, 56, 58]
129/Sv; Swiss	R170C murine *Notch3*	K-in			100%	±	+	+	–	+	+	[42, 67]
C57BL/6	R182C human *NOTCH3*	Tg		BAC	100% 150% 200% 350%	+	+	–	–	–	–	[65, 66]
C57BL/6	C455R human *NOTCH3*	Tg	SM22α		100%	+	+	+	–	–	–	[60, 62]
C57BL/6	R1031C human *NOTCH3*	Tg	SM22α		100%	+	+	+	–	–	–	[60, 62]

*KO* knock-out, *K-in* knock-in, *Tg* transgenic

### In Vitro Models

The first in vitro studies were based on primary cell models, which included VSMCs [68, 69], skin fibroblasts [70], and myoblasts [71] derived from CADASIL patients; HS683 oligodendrocytes transfected with mutant *NOTCH3* [72]; human embryonic kidney 293 cells (HEK293) and neuroblastoma cells (SH-SY5Y) in which the expression of mutant *NOTCH3* was inducible using the tetracycline (Tet)-on regulatory system [15, 73–77]; primary mouse embryonic fibroblasts (MEFs) from mice carrying mutant *NOTCH3* transgenes [78]; and Lec1-Chinese hamster ovary (CHO Lec1) cell lines transfected with plasmids encoding murine *Notch3* fragments bearing CADASIL-like mutants [78].

The possibility to induce pluripotent stem cells (iPSCs) from blood cells and skin fibroblasts has recently transformed the field of disease-specific in vitro models. iPSCs reproduce main features of embryonic stem cells and can be differentiated in different cell types [79]. The first human iPSC line from a CADASIL patient (IDISi001-A) was generated by Fernández-Susavila et al. in 2018 [80], starting from peripheral blood mononuclear cells of a patient carrying the R1242C *NOTCH3* mutation. iPSCs from a R1076C CADASIL patient were subsequently differentiated in VSMCs, embryonic, and mesenchymal stem cells by Ling et al. in 2019 [81], thus representing all three layers of vessel wall. The authors reported several aberrations of VSMCs structure and function, including increased proliferation rate and altered cytoskeleton features [81]. Notably, they described constitutive activation of NOTCH signaling, responsible for NF-κB pathway stimulation [81] consistently with previous works [82–84]. NF-κB regulates expression of key components of the inflammatory response, such as cytokines, chemokines, and adhesion molecules, but also of genes implicated in extracellular matrix remodeling [85–89], whose upregulation in CADASIL VSMCs was demonstrated by transcriptomic analysis and resulted in vascular dysfunction [81]. These findings were even more relevant in case of TNFα-induced inflammatory stimuli, suggesting an excessive cerebral blood vessels susceptibility to damage within an inflammatory scenario [81]. Furthermore, NF-κB induces expression of vimentin [90], a component of intermediate filaments, whose structure is altered in CADASIL VSMCs. Abnormalities were also detected in microfilament structure of CADASIL VSMCs, already reported in CADASIL mice and primary VSMCs from patients [31, 69], and probably associated with NOTCH3-mediated expression of smooth muscle α-actin and Rho kinase [91, 92]. Apparently, endothelial cells showed only altered innate immunity and cellular adhesion and upregulated target genes of NF-κB in presence of TNFα-induced inflammatory factors [81]. On the other side, the adventitia presented only an age-dependent loss of mesenchymal stem cells [81]. In another work, Kelleher and colleagues [93] generated iPSCs from two CADASIL patients, respectively, with R153C and C224T *NOTCH3* mutations, subsequently differentiated in vascular mural cells. iPSC-derived vascular mural cells represent an in vitro pericyte-like model, as demonstrated by the expression of pericyte markers like platelet-derived growth factor receptor beta (PDGFRβ) and NG2 [94] and by their capillary supporting function. PDGFRβ and VEGF are known stabilizing factors for microvessels [95–97]. Consequently, decreased PDGFRβ and reduced secretion of VEGF found in iPSC-derived vascular mural cells appear involved in impaired stabilization of capillary structures, partially rescued by the administration of VEGF and by siRNA knockdown of *NOTCH3*, supporting the hypothesis of a gain-of-function mechanism [93]. On the contrary, Yamamoto et al. [98] described an increased level of PDGFRβ in iPSC-derived vascular mural cells from three CADASIL patients with R182C, R41C, and C106R *NOTCH3* variants, which was reduced by DAPT, a NOTCH3 pathway inhibitor. Discrepancies across studies might be the consequence of differences in differentiation protocols, particularly because platelet-derived growth factor-BB (PDGF-BB) suppresses the expression of PDGFRβ. Anyway, the different expression of PDGFR-β, either incremented or reduced, destabilizes the structure of new blood vessels, resulting in an increased susceptibility to mechanical stress. Furthermore, iPSC-derived vascular mural cells show NOTCH3^ECD^ accumulation, which co-localize with latent transforming growth factor β binding protein-1 (LTBP-1) and HTRA1, known components of GOM [99–101].

Even though in vitro models represent a promising approach to investigate diseases’ pathogenesis, limitations associated with cell lines and iPSCs artificial reprogramming and transcriptional, morphological, and functional modifications in cell culture should be outlined. As a consequence, definite conclusions are hardly reached by in vitro models, at least without discoveries validation by in vivo approach.

## Development of Therapeutic Strategies

**Fig. 1:**
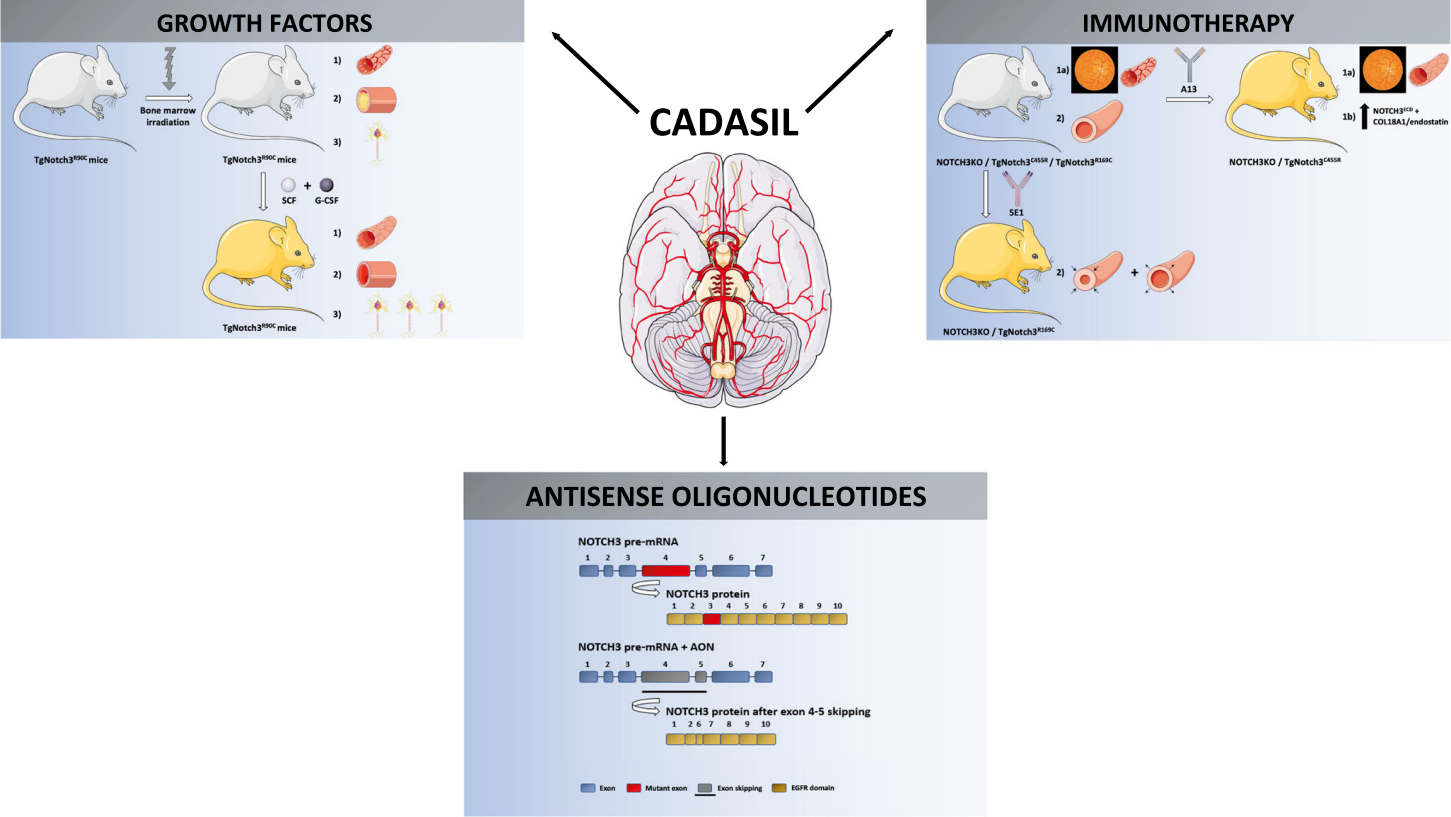
Novel therapeutic approaches under evaluation by in vitro and in vivo models. *Growth factors*: the administration of hematopoietic growth factors (SCF and G-CSF) in TgNotch3^R90C^ mice, after irradiation of bone marrow, is effective in (1) preventing VSMCs degeneration in small arteries and loss of cerebral capillaries by inhibition of apoptotic cascade; (2) reducing cerebral thrombosis; and (3) implementing neurogenesis, especially in the subventricular zone. *Immunotherapy*: agonist antibodies (A13 and 5E1) targeting NOTCH3 domains have been tested in NOTCH3KO and transgenic mice (respectively TgNotch3^C455R^ and TgNotch3^R169C^), showing respectively (1a) increased SMA coverage in retinal arterioles; (1b) increased NOTCH3^ECD^/COL18A1/endostatin levels; and (2) restoration of adequate vasodilatory responses and myogenic tone. *AONs*: exon skipping through AONs excludes mutant EGFRs with no impairment of NOTCH3 structure or function and produces a novel “EGFR fusion domain,” with correctly spaced cysteines, derived from parts of EGFR domains encoded by exons located before and after those skipped through AONs (except for exon 2–3 skipping)

To date, only few therapeutic approaches are applied in clinical practice for CADASIL. Hereafter, after a brief overview of these common strategies in CADASIL, we focus the review on novel therapeutic approaches, such as immunotherapy, growth factors administration, and antisense oligonucleotides, which are currently under investigation and are illustrated in Fig. 1.

### Pharmacological Approaches and Control of Risk Factors

#### Stroke

A single report of thrombolysis for stroke in a CADASIL patient has been reported [102], without sign of intracranial hemorrhages, a feared side effect in this population known to have microbleeds [9, 103]. Similarly, the benefit of antiplatelet drugs in CADASIL ischemic stroke prevention is unclear [104], and cases of aspirin-associated intraparenchymal bleeding are described [105]; despite of this uncertainty, most neurologists administer aspirin or clopidogrel, according to the guidelines applied for sporadic stroke. The usual prevention measures employed for stroke are applied also in CADASIL patients, including avoiding cigarette smoking, because of its relation with an earlier age of onset and incremented risk of stroke and migraine [106, 107]; managing hypertension, trying not to reach excessively low blood pressures, which are associated with an increased risk of dementia [108, 109]; and controlling diabetes [110], alcohol intake, obesity, and hypercholesterolemia with the help of statins. As above reviewed, chronic cerebral hypoperfusion might be involved in ischemic events in CADASIL patients; however, a few trials targeting vascular dysfunction failed to reveal any benefit of atorvastatin [111] and sapropterin, which acts as cofactor in nitric oxide synthesis [112]. After successful results in small studies [44, 113, 114], further investigations are required to demonstrate clinical benefits of acetazolamide and lomerizine.

#### Migraine

Common analgesic medications can be employed during CADASIL migraine attacks, especially a fixed combination of acetaminophen, acetylsalicylic acid, and caffeine [115]. Anecdotal reports describe also effectiveness of sodium valproate in acute setting [116]. Safety of triptans and ergot derivatives should be demonstrated before their use is implemented in clinical practice, due to the risk of vasoconstriction and injury of capillary endothelium [117, 118]. Many anecdotal reports have recorded a quite striking effect of acetazolamide for migraine prophylaxis with ensuing hypothesis on this drug way of action in CADASIL [119]. Amitriptyline, beta-blockers, flunarizine, and topiramate should be used with caution because of their impact on mood and cognitive disturbances [120]. Detection of higher homocysteine levels in CADASIL patients with migraine, especially in case of early age of onset [121], suggests the possibility to employ homocysteine lowering therapies with B vitamin supplementation to reduce migraine severity and frequency [122], but further investigations are warranted.

#### Cognitive Impairment

The identification of cholinergic deficit in CADASIL [123, 124] led to start clinical trials aimed to test acetylcholinesterase inhibitors. The use of donepezil in a multicenter randomized controlled trial with 168 patients did not show to improve the primary endpoint (the Vascular Dementia Assessment Scale cognitive subscale) significantly but produced some differences in secondary endpoints concerning executive function [125]. However, the presence of a small number of patients with dementia and the brief follow-up suggests that, again, larger experiences are needed [126].

#### Psychiatric Disturbances

Psychiatric symptoms seem to respond to common treatments used in sporadic disturbances. Anecdotal cases described the efficacy of quetiapine in a bipolar disorder [127] and of risperidone, sodium valproate, and flupentixol in schizophreniform organic psychosis [128].

### Immunotherapy

Recently, NOTCH3KO and TgNotch3^C455R^ mice were used to assess the impact of agonist antibodies (A13) targeting the NOTCH3 receptor’s negative regulatory region (NRR) located in the extracellular domain on preventing mural cell loss, a pathological hallmark of SVDs involved in CADASIL pathogenesis [129]. NRR stabilizes noncovalent bonds, thus maintaining NOTCH3 in an autoinhibited configuration in the absence of the ligand (Delta or Jagged). A S2 cleavage site, which undergoes ADAM-mediated proteolysis after the interaction of NOTCH3 with its ligands, is located within the NRR [130]. As above mentioned, unlike other *NOTCH3* mutations, C455R impairs ligand-mediated NOTCH3 signaling [60]. Authors chose to evaluate mural cell coverage of retinal vessels, due to their blood barrier similarities with the cerebral one, by α-smooth muscle actin (SMA) staining, whose expression is not influenced by NOTCH3 activity modifications [129]. Firstly, after ascertaining a severe reduction of mural cell coverage in retinal vessels of NOTCH3KO and TgNotch3^C455R^ mice of 6 months of age, authors observed that expressing WT human *NOTCH3* transgene, but not C455R *NOTCH3* transgene, was able to avoid mural cell loss both in NOTCH3KO and in TgNotch3^C455R^ mice [129]. In vitro the A13 antibody succeeded in activating not only the WT but also the C455R mutant receptor, by exposing the S2 cleavage site and subsequently destabilizing the NRR independently from the Jagged 1 ligand [129]. Simultaneously, NOTCH3^ECD^, which is known to be reduced in TgNotch3^C455R^ mice [62], was increased in the cell culture supernatant of WT and C455R cells incubated with A13, and this result was not affected by the addition of compound E, an A13 inhibitor, even though NOTCH3 signaling was reduced [129]. The effect of agonist antibody was investigated in vivo by injecting A13 in TgNotch3^C455R^ mice for 5 weeks after the 8th day of life. At 6 weeks of age, SMA coverage in retinal arterioles was duplicated, whereas no differences were observed in large retinal arteries, thus suggesting a NOTCH3-mediated mural cell loss prevention [129]. Concurrently, NOTCH3^ECD^ and COL18A1/endostatin levels were found increased as consequence of NOTCH3 signaling activation, unlike HTRA1 and insulin-like growth factor binding protein-1 (IGFBP-1) [129]. This finding suggests a potential role of NOTCH3^ECD^ and COL18A1/endostatin plasma levels as potential biomarkers of NOTCH3 activity.

In a following work, another antibody targeting the extracellular domain of NOTCH3 (5E1) was chronically administered to TgNotch3^R169C^ mice [131], which show the main pathological features of CADASIL, as previously mentioned [34]. The results consisted of restoration of regular vasodilatory responses and myogenic tone, without effect on NOTCH3^ECD^ and GOM deposition even if therapy was begun before the achievement of a plateau of deposits [131]. Compared to Alzheimer’s disease mouse models, in which extracellular ß amyloid plaques disappear after treatment with antibodies against amyloid ß peptide, perhaps as consequence of microglial and macrophage activation [132–134], in CADASIL mice microglial might be dysfunctional and so unable to remove NOTCH3^ECD^ and GOM deposits. An alternative hypothesis suggests that brain dysfunction might be related to soluble species rather than aggregates. In the previously reported experiments, 5E1 might have acted by eliminating these soluble toxin assemblies or by delaying their release from extensive aggregates [131].

Even though the potential of immunotherapy appears intriguing, further investigations and considerable improvements are required in vivo before moving to humans. First, the choice of antibodies should consider the affinity for NOTCH3 receptor and the ability to cross the blood–brain barrier, thus allowing to reach the brain in a more elevated percentage when peripherally administered; secondly, mice with pre-existing cerebrovascular features should be involved, thus providing a complete therapeutic context [131].

### Growth Factors

Different works recently showed a potential benefit of administering hematopoietic growth factors such as stem cell factor (SCF), granulocyte colony-stimulating factor (G-CSF), and their combination in the acute, subacute, and even chronic phase of ischemic stroke in animal models [135–138]. SCF and G-CSF are deeply involved in blood cell generation as well as bone marrow cell renewal and mobilization [139]. Starting from these evidences, Liu et al. [140] tried to replicate these positive results in a CADASIL mouse model (TgNotch3^R90C^ mice), which was irradiated to destroy bone marrow at 8 months, transplanted with the bone marrow of UBC-GFP transgenic mice for tracking bone marrow-derived cells in the brain, and treated with recombinant mouse SCF and recombinant human G-CSF after a 1-month recovery. The results were promising. Compared to control group, the treated arm displayed a long-term improvement of cognitive function in terms of spatial learning and memory, which are severely affected in TgNotch3^R90C^ mice, as demonstrated by the water maze test performed at 11 and 19 months of age [140]. This effect might be related to the evident prevention of VSMCs degeneration in small arteries and loss of cerebral capillaries, whose pathogenic role in CADASIL evolution is well established [34, 37–39, 44, 141, 142], in addition to the inhibition of GOM formation. The explanation for this finding is not fully clear, even though it is thought to be related to VSMCs survival via AKT pathway and inhibition of caspase-3 cascade during an exposure to antiapoptotic factors. The authors highlighted the presence of altered capillaries in the brains of CADASIL mice made of bone marrow-derived endothelial cells [140]. This finding would explain the continuous consumption of endothelial progenitor cells observed in CADASIL patients, as consequence of their role in the replacement of damaged endothelium in course of vascular degeneration [143]. These abnormal capillaries are decreased in brains of SCF + G-CSF-treated CADASIL mice [140]. Furthermore, treatment with SCF + G-CSF was found to prevent the apoptosis cascade in mice; to implement neurogenesis, specifically in the subventricular zone, perhaps as consequence of the reduced loss of neural stem cells and neural progenitor cells compared to CADASIL mice, which are probably involved in vascular cells renewal in conditions of VSMCs degeneration, brain capillaries damage, and chronic brain ischemia; and to expand the number of bone marrow-derived cells especially in the hippocampus [140], which are thought to differentiate in neuronal-like cells in the brain, as consistent with previous works [135, 136, 144].

Subsequently, the same group repeated experiments on TgNotch3^R90C^ mice to assess the impact of SCF + G-CSF administration at 9, 10, 12, 15, and 20 months of age on cerebral thrombosis occurrence [145]. The higher rate of thrombosis with associated IgG extravasation was detected in small arteries and in cerebral capillaries, especially near bifurcations, which represent areas of complex blood flow, predisposed to injury [145]. As strictly linked to this evidence, the authors described endothelial cell loss and replacement by bone marrow-derived cells all over the thrombotic regions [145], as previously reported in a mouse model of ischemic stroke [146]. However, these novel cerebral vessels show blood–brain barrier leakage, thus suggesting a potentially dysfunctional nature. Small vessel thrombosis appeared deeply reduced in the treatment group at 22 months of age compared to the control one [145].

VEGF and VEGF-A have been found to be notably reduced in TgNotch3^R90C^ mice brains of 10–11 months of age compared to aged-matched WT controls [147]. Their role in angiogenesis is well known [148, 149]. Even though the relation between *NOTCH3* mutations and decreased VEGF and VEGF-A levels is undetermined, the result is a reduction in cerebral vessel density, significantly associated with the reduction of dendrites, axons, and synapses in the somatosensory and motor cortex layer 2/3 and in the hippocampal CA1, and decreased neurogenesis in the subventricular zone and subgranular zone, resulting in impaired spatial learning and memory [147], as previously reported [140]. The administration of SCF + G-CSF at 9 and 10 months of age, before the occurrence of pathological alterations in VSMCs of TgNotch3^R90C^ mice [37], was able to prevent all these processes, thus improving cognitive function in TgNotch3^R90C^ mice. Since the use of Avastatin, a VEGF-A inhibitor, was able to impede the pro-angiogenetic and neurogenetic effect of SCF + G-CSF, the authors hypothesized that VEGF-A was required for SCF + G-CSF-mediated brain repair. Indeed, the administration of SCF + G-CSF in TgNotch3^R90C^ mice enhanced VEGF and VEGF-A levels. In agreement with findings of previous studies in experimental models of chronic stroke [150, 151], the potential role of NF-κB, a transcription factor which induces VEGF expression and angiogenesis [152, 153], on SCF + G-CSF-enhanced cerebral VEGF and VEGF-A production needs to be clarified.

Even though subcutaneous injection of cell growth factors appears a suitable approach and has been already employed for bone marrow recovery after chemotherapy in patients with cancer, the beneficial effects in CADASIL patients remain largely unknown, thus requiring further investigations prior to human administration.

### Antisense Oligonucleotides

The dramatic impact of small synthetic RNA or DNA oligomers to bind specific sequences of pre-mRNA of target genes, thus excluding specific exons from splicing and, consequently, from the mature mRNA, has been already highlighted for monogenic neurologic diseases, such as Duchenne muscular dystrophy [154] and spinal muscular atrophy [155].

Tikka et al. [69] obtained *NOTCH3* silencing by employing short hairpin RNA (shRNA) in CADASIL and control VSMCs to assess the aforementioned vasoregulatory role of NOTCH3. The result was a statistically significant reduction of NOTCH3 target HES5 expression and signaling, as well as actin aberrations, both in VSMCs from controls and CADASIL patients [69].

In 2016, Rutten et al. [156] provided a first experimental exon skipping approach for CADASIL to prevent NOTCH3^ECD^ toxic accumulation in vitro. Even though it could be used for 18 of 23 exons encoding for EGFR domains, embracing 94% of all CADASIL mutations, the authors selected antisense oligonucleotides (AONs) to skip specifically exons 2–3, 4–5, and 6, based on *in silico* predictions of the effect of EGFRs exclusion on primary, secondary, and tertiary structure. As expected, mutant EGFR was excluded with no impairment of NOTCH3 structure or function. Except for exon 2–3 skipping, the idea is not only to eliminate the mutant EGFR domain but also to realize a novel “EGFR fusion domain,” with six correctly spaced cysteines, derived from parts of EGFR domains encoded by exons located before and after those skipped through AONs. The result is a shorter NOTCH3 protein with no pathogenic unpaired cysteines. Skip proteins were proved to have a normal subcellular localization in transfected fibroblasts, predominantly perinuclear, reflecting an accumulation in RE and Golgi network [73, 74], and secondary at the cell surface where they co-localized with the ligand jagged 1, ensuring a correct protein processing and ligand-binding ability. Furthermore, a luciferase report assay confirmed a conserved binding activation rate. Subsequently, exon skipping revealed to be successful also in patient-derived VSMCs, and, at the same time, a reduction of *NOTCH3* and downstream target genes (*HES1*, *HEYL*, *PDGFR-β* and *JAG1*) expression was excluded. However, the consequences of exon skipping on NOTCH3 aggregation in vessels, such as NOTCH^ECD^ deposits, GOM and VSMCs loss, could not be assessed because it requires in vivo studies, thus highlighting the importance of mouse models. A potential alternative to exon skipping could be *NOTCH3* downregulation through AONs inducing RNase H-mediated degradation of RNA, something that remains to be done [156].

The importance of exon skipping as therapeutic approach in CADASIL has been recently suggested by the detection of a *NOTCH3* mutation (G498C), which induces exon 9 skipping due to its location in a donor splice site, in a 63-year-old proband and in three siblings showing only WMH on MRI and no other CADASIL neuroradiological features. Overall, they presented a mild phenotype, with non-migraine headaches or initial cognitive impairment, and familiarity for ischemic stroke. Skin biopsies failed to reveal GOM and showed only mild NOTCH3^ECD^ accumulation. In this case, transfected fibroblasts showed normal localization of NOTCH3 protein at the cell surface, but impaired ligand-dependent signaling. To confirm the translatability of their finding, the authors realized both an exon skipping by AONs in transfected VSMCs and a gene editing through CRISPR/Cas9 in HEK293 cells [157].

Obviously, in vitro studies represent only the first step in the process of validation of cysteine corrective exon skipping as efficacious therapeutic approach for CADASIL. In vivo studies on animal models are required before moving to the human setting. However, these encouraging preliminary data seem to extend the growing list of potential therapeutic applications of exon skipping [158].

## Conclusions

CADASIL is a unique model of SVDs, with typical clinical and neuroradiological features as well as pathological hallmarks represented by NOTCH3 accumulation and GOM deposits in abnormal VSMCs. Despite the efforts, the well-known genetic landscape and the growing number of reliable disease models, its pathogenesis and the specific NOTCH3 pathway involved remain elusive. In vivo models, especially knock-out, knock-in, and transgenic mice, have provided a large amount of data, expanding our molecular and biological knowledge of underlying pathogenic mechanisms which involve altered myogenic responses secondary to a potassium channelopathy-like defect, which result in impaired cerebral blood flow autoregulation, hypoperfusion, and ischemia susceptibility. The most evident limit of mouse models is surely represented by the lack of pathological and clinical CADASIL features, thus making necessary the development of different disease models. Over the last years, iPSC technology has revolutionized the field of in vitro models. The possibility to investigate disease underlying processes and to test novel therapeutic approaches in a safe and truthful manner is opening new unexplored avenues for so many neurological disorders. In this scenario, available therapeutic strategies for CADASIL remain inadequate. Novel approaches have been recently tested in vitro and in vivo, including immunotherapy [129, 131], growth factors administration [140, 145, 147], and exon skipping through AONs [156]. All these techniques have shown promising results in preliminary studies and have been already employed as revolutionary therapy for other neurological and non-neurological diseases [154, 155]. To prevent side effects of novel therapies, alterations of NOTCH3 signaling involved in CADASIL pathogenesis, which are still uncertain, should be cautiously evaluated. Even though the feeling of lack of knowledge remains, we are moving to an increased availability of potential therapeutic targets, so that we can hope for phase 1 clinical trials in the next future.

## Data Availability

Not applicable.
